# Public Knowledge, Practices, and Acceptance of Smart Health Technologies Addressing Antibiotic Use and Resistance in Saudi Arabia

**DOI:** 10.3390/healthcare14162480

**Published:** 2026-08-11

**Authors:** Nahed N. Mahrous, Samia T. Al-Shouli, Yousef M. Hawsawi, Mohammed Aljuwayd, Abdullah AlDhuwaihy, Abdulrahman AlOmar, Yahya F. Jamous

**Affiliations:** 1 Department of Biology, College of Science, University of Hafr Al-Batin, Hafar Al-Batin 39524, Saudi Arabia; 2 Immunology Unit, Department of Pathology, College of Medicine, King Saud University, Riyadh 11461, Saudi Arabia; salshouli@ksu.edu.sa; 3 Research Center, King Faisal Specialist Hospital and Research Center, P.O. Box 40047, Jeddah 23433, Saudi Arabia; hyousef@kfshrc.edu.sa; 4 Department of Biochemistry and Molecular Medicine, College of Medicine, Alfaisal University, P.O. Box 50927, Riyadh 11533, Saudi Arabia; 5 Medical Laboratory Technology, College of Applied Medical Science, Northern Border University, Arar 73213, Saudi Arabia; mohammed.aljuwayd@nbu.edu.sa; 6 College of Medicine, King Saud University, Riyadh 11461, Saudi Arabia; dradhuwaihy@gmail.com (A.A.); mrdhomd@gmail.com (A.A.); 7 Wellness and Preventive Medicine Institute, Health Sector, King Abdulaziz City for Science and Technology (KACST), Riyadh 11442, Saudi Arabia

**Keywords:** antimicrobial resistance, antibiotic resistance, antibiotic use, smart health technology, digital health platforms, antibiotic stewardship, health communication

## Abstract

**Background:** Antimicrobial resistance (AMR) remains a major global public health threat, largely driven by inappropriate antibiotic use. Smart health technologies (SHTs), including mobile health applications and digital stewardship tools, offer promising opportunities to improve antibiotic use and strengthen antimicrobial stewardship. This study assessed public knowledge, practices, and stated acceptance of selected SHTs related to antibiotic use and AMR management among adults residing in Saudi Arabia. **Methods:** A cross-sectional online survey was conducted among adults residing in Saudi Arabia between November 2025 and January 2026 using a structured questionnaire assessing knowledge and practices related to antibiotic use, awareness of SHTs, perceived barriers, and acceptance of digital health technologies. Descriptive and inferential statistical analyses were performed. **Results:** A total of 434 consenting participants were included in the final analysis. Participants demonstrated gaps in knowledge of antibiotic use and AMR, while misconceptions and inappropriate practices remained common, including lifetime non-prescription antibiotic use (37.8%) and sharing antibiotics (31.3%). Awareness of SHTs was limited, with only 30.4% aware of antibiotic-related mobile health applications. Despite limited awareness, 61.8% were willing to use medication-reminder applications and 87.3% preferred electronic prescriptions. The mean acceptance score was 4.39 ± 0.66/5, and 79.7% had high stated acceptance. In an exploratory adjusted analysis, female respondents had higher odds of meeting the predefined high-acceptance threshold (AOR = 1.88, 95% CI: 1.04–3.40); however, this finding should be interpreted cautiously because the overall regression model was not statistically significant and showed modest discrimination. Perceived barriers were not independently associated with acceptance. **Conclusions:** Despite gaps in knowledge and persistent inappropriate antibiotic-use practices, the surveyed online respondents demonstrated favorable stated attitudes toward selected smart health technologies. These findings should not be interpreted as evidence of actual adoption or national implementation readiness. Further nationally representative and prospective studies are required before broader implementation can be recommended.

## 1. Introduction

Antimicrobial resistance (AMR) is a major threat to global health, health security, and sustainable development in the twenty-first century [1,2]. AMR is associated with significant morbidity and mortality worldwide. An estimated 4.95 million deaths were associated with bacterial AMR in 2019, with 1.27 million deaths directly attributed to bacterial AMR [3]. Recent projections indicate that, if current trends persist, AMR-related mortality will continue to rise substantially over the coming decades, potentially causing 10 million preventable deaths annually by 2050 [4]. Beyond its clinical consequences, AMR imposes a substantial economic burden through increased healthcare expenditures, prolonged hospitalizations, and productivity losses. According to the World Bank, AMR was estimated to cost an additional USD 1 trillion in healthcare expenditures by 2025 and cause annual gross domestic product (GDP) losses of between USD 1 and 3.4 trillion by 2030 [5]. Although antimicrobial resistance is driven by biological adaptation, its emergence and spread are largely shaped by human behaviour. Inappropriate prescribing, self-medication, non-adherence to treatment regimens, over-the-counter access to antibiotics, sharing of medications, and misuse of antimicrobials in agriculture and animal production create selective pressures that accelerate the development and dissemination of resistant pathogens [6,7]. Consequently, AMR is increasingly recognized as a preventable and behavior-driven public health challenge. Technology-enabled approaches may complement conventional education, regulation, surveillance, infection prevention, and antimicrobial stewardship by supporting medication management, timely communication, and evidence-based antimicrobial use [8,9].

The burden of AMR is significant in the Middle East region [10,11,12]. High rates of antibiotic consumption, widespread self-medication practices, and inappropriate antibiotic use have been reported in several countries in the region [13]. Contributing factors to the increased burden of AMR in the Middle East include easy access to antibiotics without prescription, inadequate enforcement of pharmaceutical regulations, variable antimicrobial stewardship implementation, population mobility, humanitarian crises in some countries, and limited public understanding of antimicrobial resistance [14]. These challenges are compounded by healthcare-system pressures and growing rates of multidrug-resistant organisms, making AMR a significant regional public health priority [15]. As a result, the region continues to experience increasing levels of resistance among common bacterial pathogens, threatening the effectiveness of currently available antimicrobial therapies.

Saudi Arabia represents a particularly important setting for addressing AMR because of its unique demographic, healthcare, and epidemiological characteristics. Antibiotics account for a substantial proportion of prescribed medications within the Kingdom, while the country experiences high levels of international travel and population movement associated with tourism, expatriate communities, and religious mass gatherings. Studies have documented the presence of important resistant pathogens, including methicillin-resistant *Staphylococcus aureus* (MRSA), extended-spectrum β-lactamase-producing organisms, and carbapenem-resistant bacteria [16]. Saudi Arabia has introduced prescription-only antibiotic-dispensing regulations, national AMR action plans, antimicrobial stewardship activities, public-awareness initiatives, infection-prevention programmes, and resistance-surveillance efforts. Despite these measures, previous studies have reported misconceptions regarding the effectiveness of antibiotics against viral infections, premature discontinuation of treatment once symptoms improve, storage and reuse of leftover antibiotics, and sharing of antibiotics among family members [17,18,19]. These findings indicate that persistent gaps in public knowledge and practices may require complementary, rather than replacement, approaches within the broader national AMR strategy.

In this study, smart health technologies (SHTs) refer to selected digital tools intended to support medication management, medication adherence, antibiotic-related information, and responsible antibiotic use. These include mobile medication-reminder applications, SMS alerts, smart-device reminders, electronic prescriptions, and mobile health applications providing antibiotic-related information or guidance. Compared with conventional one-directional approaches such as posters, television campaigns, websites, and general social-media messages, selected SHTs may offer more individualized and interactive functions, including real-time reminders, tailored information, bidirectional communication, integration with electronic prescriptions, adherence support, and linkage with healthcare services [20,21,22]. Emerging evidence suggests that digital tools can support more appropriate antimicrobial prescribing, improve public awareness of AMR, enhance surveillance systems, and strengthen stewardship programmes across healthcare settings [23]. However, these potential benefits depend on usability, accessibility, sustained engagement, privacy protection, and integration with existing healthcare systems, and they should not be assumed solely from favorable survey responses.

The growing emphasis on digital transformation within Saudi Arabia further strengthens the relevance of technology-based approaches to AMR control [24]. Through Saudi Vision 2030, the Kingdom has prioritized digital health innovation, healthcare modernization, artificial intelligence adoption, and improved population health outcomes. National initiatives have expanded the use of electronic health records, telehealth services, mobile health applications, and integrated digital healthcare platforms [25]. These developments create a favourable environment for evaluating SHTs that may support antimicrobial stewardship, strengthen public awareness, and encourage responsible antibiotic use. Such technologies should be viewed as complementary to antibiotic-access regulation, antimicrobial-consumption and resistance surveillance, hospital stewardship programmes, professional education, infection prevention and control, and sustained public education. Integrating AMR-prevention strategies within the broader digital-health agenda aligns with national healthcare-transformation objectives and offers an opportunity to leverage existing digital infrastructure to address a major public-health challenge [26].

Despite increasing recognition of the potential of SHTs, limited evidence exists regarding public awareness, perceptions, and stated willingness to use selected digital tools for antibiotic stewardship and antimicrobial-resistance management in Saudi Arabia. Understanding these attitudes may help inform the design of future user-centred interventions. However, stated willingness should not be interpreted as actual adoption, sustained use, implementation readiness, or demonstrated effectiveness. This study aimed to assess public knowledge, practices, awareness, perceived barriers, and stated acceptance of selected smart health technologies related to antibiotic use and antimicrobial resistance among adults residing in Saudi Arabia.

## 2. Methodology

### 2.1. Ethical Considerations

Ethical approval was obtained from the Research Ethics Committee at King Abdulaziz City for Science and Technology (KACST) before data collection (approval no. 25019; 27 August 2025). Informed consent was obtained from all participants, and participation was voluntary. The confidentiality of participants’ responses was maintained throughout the study. The research team complied with the Saudi Regulations of Research Bioethics on Living Creatures. No invasive procedures or clinical interventions were conducted.

Adult participants aged 18 years or older were eligible if they resided in Saudi Arabia, were able to read and understand Arabic or English, and provided informed consent. Eligible participants included members of the general public and healthcare and non-healthcare professionals. Individuals were excluded if they were younger than 18 years, did not reside in Saudi Arabia, were unable to read Arabic or English, or did not provide informed consent.

### 2.2. Study Design

A cross-sectional survey design was used to assess public knowledge, practices, awareness, perceived barriers, and stated acceptance of selected smart health technologies (SHTs) related to antibiotic use and antimicrobial resistance among adults residing in Saudi Arabia.

In this study, SHTs were operationally defined as digital tools intended to support medication management, medication adherence, antibiotic-related information, and responsible antibiotic use. The evaluated technologies included mobile medication-reminder applications, SMS alerts, smart-device reminders, electronic prescriptions, and mobile health applications providing antibiotic-related information or guidance.

A self-administered online questionnaire was selected to collect quantitative data during the defined cross-sectional study period. Data were collected from November 2025 to January 2026. The three-month data-collection period was selected because the electronically administered questionnaire could be repeatedly circulated through the designated recruitment channels. This period was sufficient to obtain a sample exceeding the minimum calculated requirement and was appropriate for the cross-sectional study design.

A convenience-sampling approach was used. The questionnaire was distributed electronically through WhatsApp and email networks, and the survey link was shared with eligible adults residing in different regions of Saudi Arabia. Participation was voluntary, and no personally identifying information was collected. Because the questionnaire link was openly distributed and no defined sampling frame was available, the total number of individuals who received or viewed the invitation could not be determined; therefore, a conventional response rate could not be calculated.

A total of 450 entries were recorded. The first 10 entries corresponded to the preliminary pilot assessment conducted before the formal survey launch. These entries were used to evaluate questionnaire clarity, wording, response options, survey flow, and approximate completion time. Minor wording and formatting refinements were made based on the pilot feedback. Because these preliminary entries were recorded before the finalized informed-consent field was included, they were excluded from the analytic dataset and were not included in any statistical analysis. Among the subsequent 440 respondents, six did not provide consent and were also excluded. The final analytic sample therefore consisted of 434 consenting participants. The survey platform required responses to all mandatory items before submission; consequently, no item-level missing data were present among the included responses.

The questionnaire was developed based on published studies examining public knowledge and practices concerning antibiotic use and antimicrobial resistance in Saudi Arabia, together with World Health Organization public-awareness guidance and survey materials. The questionnaire was available in Arabic and English, with equivalent items and response options in both versions. The two language versions were internally reviewed by members of the research team to assess consistency in meaning, clarity, and wording before distribution. A preliminary pilot assessment involving 10 responses was conducted to evaluate questionnaire clarity, wording, response options, survey flow, and approximate completion time. Minor wording and formatting refinements were made based on the feedback received. Formal forward–backward translation procedures, external content-validity indices, construct-validity testing, and domain-specific internal-consistency coefficients were not documented. Therefore, the unsupported Cronbach’s alpha statement included in the original manuscript was removed. The complete questionnaire is provided as Supplementary Material S1.

The questionnaire was divided into five sections covering demographic characteristics; knowledge and practices related to antibiotic use and antimicrobial resistance; awareness and previous use of selected SHTs; perceived barriers to their use; and stated acceptance of SHTs. Demographic variables included age group, gender, nationality, region of residence, educational level, employment status, and formal education or training in a healthcare-related field.

Income, presence of children, and chronic-disease status were not included in the final distributed questionnaire and were therefore removed from the description of the collected variables.

The questionnaire did not formally assess validated Technology Acceptance Model or Unified Theory of Acceptance and Use of Technology constructs. Accordingly, statements indicating that TAM or UTAUT constructs were measured or tested were removed. Acceptance was evaluated using four five-point Likert items assessing the perceived potential of smart technologies to reduce antibiotic misuse, support for their integration into public-health campaigns, willingness to modify antibiotic-use behavior with digital support, and trust in mobile health applications.

### 2.3. Sample Size Calculation

The minimum required sample size was estimated using a 95% confidence level, a 5% margin of error, and an anticipated population proportion of 50%. The 50% proportion was selected because it provides the most conservative estimate when the expected population proportion is unknown. The calculated minimum sample size was 385 participants. The final analytic sample of 434 consenting participants exceeded this requirement.

### 2.4. Statistical Analysis

Data were coded and analyzed using SPSS version 27. Data cleaning and validation were performed in Microsoft Excel before statistical analysis. Categorical variables were summarized using frequencies and percentages, whereas continuous composite measures were summarized using means, standard deviations, and observed ranges. Distributional characteristics were evaluated using histograms, Q–Q plots, and measures of skewness.

Correct knowledge responses were coded as 1, whereas incorrect and “not sure” responses were coded as 0. Higher knowledge scores indicated greater knowledge of antibiotic use and antimicrobial resistance. Practice items were coded so that safer reported behaviors received a score of 1 and potentially inappropriate behaviors received a score of 0. Higher practice scores therefore indicated safer reported antibiotic-use practices.

Awareness of antibiotic-related mobile applications, previous use of digital reminders, willingness to use reminder applications, and preference for electronic prescriptions were analyzed and reported separately because they represented conceptually distinct outcomes.

Each reported barrier was coded as 1 and summed to produce a barrier count ranging from 0 to 5, with higher values indicating a greater number of perceived barriers. Respondents who selected “nothing” received a barrier count of 0.

The acceptance score was calculated as the mean of four five-point Likert items. Responses were coded from 1 for “strongly disagree” to 5 for “strongly agree,” producing a score ranging from 1 to 5. Higher scores indicated greater stated acceptance. High acceptance was defined as a mean score of ≥4, corresponding to an average response of at least “agree.” Scores below 4 were classified as lower acceptance. Because dichotomization may reduce statistical information, analyses using the continuous acceptance score were retained alongside the binary high-acceptance outcome.

Welch’s independent-samples t-test was used to compare mean acceptance scores between men and women because the group sizes and variances differed. The Kruskal–Wallis test was used to compare acceptance scores across age groups because the scores were concentrated toward the upper end of the scale. Chi-square tests were used to examine associations between categorical variables and high acceptance. Spearman’s rank correlations were used to evaluate associations between composite measures and the continuous acceptance score, with 95% confidence intervals estimated using bootstrap resampling.

Multivariable logistic regression was performed to identify factors associated with high acceptance. Age group, gender, educational level, healthcare-related education or training, and barrier count were entered simultaneously based on their conceptual relevance to digital access, health literacy, health behavior, and technology acceptance. Categorical predictors were entered using indicator coding. The reference categories were age 18–24 years, male gender, bachelor’s degree, and no healthcare-related education or training.

Multicollinearity was assessed using variance inflation factors. Model calibration was evaluated using the Hosmer–Lemeshow goodness-of-fit test, and discrimination was assessed using the area under the receiver-operating-characteristic curve. Adjusted odds ratios and corresponding 95% confidence intervals were reported. The final model included all 434 consenting participants because no item-level missing data were present in the analyzed dataset. A two-sided *p*-value of <0.05 was considered statistically significant.

## 3. Results

### 3.1. Descriptive Statistics

A total of 450 questionnaire entries were recorded. The first 10 entries corresponded to the preliminary pilot assessment conducted before the formal survey launch and were excluded from the analytic dataset. Of the subsequent 440 respondents, six did not provide consent and were also excluded. The final analytic sample therefore comprised 434 consenting participants. All mandatory questionnaire items were completed for the included participants.

The descriptive statistics of the two verified composite measures are presented in Table 1. The mean barrier count was 1.04 ± 1.06, with values ranging from 0 to 5. The acceptance score, calculated as the mean of four five-point Likert items, ranged from 1.25 to 5.00 and had a mean of 4.39 ± 0.66. A total of 346 participants (79.7%) met the predefined criterion for high stated acceptance, corresponding to a mean acceptance score of ≥4.

Knowledge and antibiotic-use practices were reported at the item level rather than interpreted using unvalidated thresholds. Awareness, previous technology use, willingness to use reminder applications, and preference for electronic prescriptions were also reported as separate outcomes because they represent conceptually distinct measures.

#### 3.1.1. Socio-Demographic Characteristics

The sociodemographic characteristics of the 434 included participants are presented in Table 2. Participants were distributed across all five age categories, with the largest group aged 36–45 years (24.4%). Most respondents were male (64.7%), Saudi nationals (97.0%), and residents of the central region (74.0%). More than half held a bachelor’s degree (52.3%), while 12.4% held a postgraduate degree. The largest employment group consisted of participants employed outside the health sector (43.1%), followed by unemployed participants (32.3%). Approximately 30.6% had received formal education or training in a healthcare-related field.

The geographic concentration in the central region and the high proportion of Saudi and university-educated respondents should be considered when interpreting the generalizability of the findings.

#### 3.1.2. Knowledge of Antibiotics and Resistance

The item-level knowledge findings are presented in Table 3. Only 161 participants (37.1%) correctly recognized that antibiotic resistance means that antibiotics cease to work effectively against the microorganism. In contrast, 136 (31.3%) incorrectly believed that the human body becomes resistant to antibiotics, 39 (9.0%) believed that antibiotics become stronger over time, and 98 (22.6%) were unsure.

Most participants identified antibiotic overuse (68.9%) and failure to complete a prescribed course (56.9%) as contributors to resistance. However, only 33.6% selected antibiotic use for viral infections, and 20.7% selected sharing leftover antibiotics as contributing factors.

Although 68.0% selected bacteria as the organisms against which antibiotics are primarily effective, substantial proportions also selected viruses (39.9%), fungi (24.9%), and parasites (17.7%), demonstrating persistent misconceptions. A total of 63.4% recognized that unnecessary antibiotic use can contribute to microbial resistance, while 72.8% correctly indicated that it is not safe to stop antibiotics solely because symptoms improve.

Overall, 80.9% believed that antibiotic resistance is increasing. However, only 45.6% correctly identified resistance as occurring when microorganisms become resistant to antibiotics, whereas 38.5% attributed resistance to the human body becoming resistant.

#### 3.1.3. Practices Related to Antibiotic Use

The reported antibiotic-use practices are presented in Table 4. A total of 164 participants (37.8%) reported having taken antibiotics without a physician’s prescription at some point, and 136 (31.3%) reported having shared antibiotics with family members or friends. Conversely, 344 participants (79.3%) reported always completing a prescribed antibiotic course.

Physicians were the most frequently reported source of antibiotic-related information (84.6%), followed by pharmacists (49.3%), the internet or social media (24.0%), and friends or relatives (15.2%).

The non-prescription-use and sharing items assessed lifetime self-reported experiences. The questionnaire did not determine when these behaviors occurred or the source from which non-prescription antibiotics were obtained.

#### 3.1.4. Awareness, Previous Use, and Willingness to Use Selected Smart Health Technologies

Awareness, previous use, stated willingness, and preference regarding selected digital tools are presented separately in Table 5. Only 132 participants (30.4%) were aware of mobile health applications designed to track antibiotic use, and 163 (37.6%) had previously used a mobile application, SMS alert, or smart device as a medication reminder.

When asked about future use, 268 participants (61.8%) stated that they would be willing to use an application that reminded them to take antibiotics on schedule, while 113 (26.0%) responded “maybe.” A total of 379 participants (87.3%) stated that they would prefer an electronic prescription over a paper prescription if both options were available. These variables represent different concepts and were not combined into a single awareness or acceptance measure. In particular, preference for an electronic prescription was treated separately from voluntary adoption of a medication-reminder application.

#### 3.1.5. Perceived Barriers to Using Health Technologies

The perceived barriers to using health technologies are presented in Table 6. A total of 165 participants (38.0%) selected “nothing,” indicating that they did not identify any of the listed barriers. The most frequently selected barrier was lack of interest (27.9%), followed by lack of awareness (25.6%), privacy or security concerns (21.0%), difficulty using technology (20.0%), and cost (9.4%).

The mean number of reported barriers was 1.04 ± 1.06, with a range from 0 to 5. Because participants could select more than one barrier, the response percentages exceed 100% when summed.

#### 3.1.6. Attitudes Toward Selected Smart Health Technologies

Participants’ attitudes toward the evaluated digital tools are presented in Table 7. A total of 415 participants (95.6%) agreed or strongly agreed that the public should be educated about the appropriate use of antibiotics. This item represented support for public education and was not included in the composite acceptance score.

A total of 390 participants (89.9%) agreed or strongly agreed that smart technologies could help reduce antibiotic misuse in the community, and 392 (90.3%) supported integrating such technologies into public-health campaigns. Additionally, 348 participants (80.2%) agreed or strongly agreed that they would change how they used antibiotics if digital tools helped them track and improve adherence, while 367 (84.6%) trusted mobile health applications to provide reliable advice regarding antibiotic use.

The latter four items were used to calculate the composite acceptance score. These findings reflect favorable stated attitudes and should not be interpreted as evidence of actual adoption, sustained use, or intervention effectiveness.

### 3.2. Inferential Analysis

#### 3.2.1. Association Between Gender and Acceptance of Smart Health Technologies

Women had a slightly higher mean acceptance score than men. The mean score was 4.47 ± 0.60 among women and 4.35 ± 0.68 among men. Welch’s independent-samples t-test indicated that this difference narrowly missed the conventional threshold for statistical significance (t = −1.96, df = 349.62, *p* = 0.051).

When acceptance was dichotomized at a mean score of ≥4, 132 of 153 women (86.3%) and 214 of 281 men (76.2%) had high stated acceptance. The crude odds of high acceptance were greater among women than men (OR = 1.97, 95% CI: 1.15–3.36; Fisher’s exact *p* = 0.013).

The continuous-score and binary analyses evaluated related but non-identical outcomes. The Welch test compared the complete distributions of the mean scores, whereas the odds ratio compared the proportions meeting the predefined high-acceptance threshold (Table 8).

#### 3.2.2. Association Between Age Group and Acceptance

The mean acceptance score did not differ significantly across the five age groups (Kruskal–Wallis H = 7.37, *p* = 0.117). Similarly, the proportion of participants with high acceptance did not differ significantly by age group (χ^2^(4) = 4.15, *p* = 0.387).

High acceptance was observed among 73.0% of participants aged 18–24 years, 80.7% of those aged 25–35 years, 84.0% of those aged 36–45 years, 82.2% of those aged 46–55 years, and 78.3% of those aged ≥56 years. These findings do not indicate that favorable stated acceptance was limited to younger participants (Table 9).

#### 3.2.3. Association Between Educational Level and Acceptance

There was no statistically significant association between educational level and high stated acceptance (χ^2^(4) = 4.27, *p* = 0.371). High acceptance was observed across all educational categories, ranging from 75.0% among high-school graduates to 93.3% among participants with less than a high-school education. The estimate for the latter category should be interpreted cautiously because it included only 15 participants (Table 10).

#### 3.2.4. Association Between Healthcare-Related Training and Acceptance

High acceptance was reported by 243 of 301 participants without healthcare-related education or training (80.7%) and 103 of 133 participants with healthcare-related education or training (77.4%). The difference was not statistically significant (χ^2^(1) = 0.43, *p* = 0.512).

#### 3.2.5. Correlation Between Perceived Barriers and Acceptance

There was no meaningful correlation between the barrier count and the continuous acceptance score (Spearman’s ρ = 0.005, *p* = 0.918). Thus, reporting a greater number of barriers was not associated with either higher or lower stated acceptance in the bivariate analysis.

### 3.3. Multivariable Logistic Regression

A multivariable logistic-regression model was fitted to examine factors associated with high stated acceptance, defined as a mean acceptance score of ≥4. The model included age group, gender, educational level, healthcare-related education or training, and barrier count. All 434 participants were included in the analysis.

After adjustment, women had higher odds of high stated acceptance than men (AOR = 1.88, 95% CI: 1.04–3.40; *p* = 0.036). None of the age categories, educational categories, healthcare-related training, or barrier count was independently associated with high acceptance. In particular, the barrier count was not associated with the outcome (AOR = 1.00, 95% CI: 0.80–1.25; *p* = 0.977).

The Hosmer–Lemeshow test indicated adequate calibration (χ^2^ = 4.93, df = 8, *p* = 0.765), and the area under the receiver-operating-characteristic curve was 0.631, indicating modest discrimination. The likelihood-ratio test for the model as a whole was not statistically significant (*p* = 0.274), with a pseudo-R^2^ of 0.030. Therefore, the adjusted association with gender should be interpreted cautiously and as exploratory rather than conclusive. Variance inflation factors were below 3, indicating no important multicollinearity among the predictors (Table 11).

## 4. Discussion

This study examined public knowledge and practices related to antibiotic use and antimicrobial resistance, together with awareness, perceived barriers, and stated acceptance of selected smart health technologies among adults residing in Saudi Arabia. The findings identified persistent misconceptions and potentially inappropriate antibiotic-use practices, including lifetime non-prescription use and antibiotic sharing. Awareness and previous use of the evaluated digital tools were limited; nevertheless, most respondents expressed favorable stated attitudes toward selected SHTs. In an exploratory adjusted analysis, women had higher odds of meeting the predefined high-acceptance threshold than men. However, this finding should be interpreted cautiously because the overall regression model was not statistically significant and demonstrated only modest discrimination. Age group, educational level, healthcare-related education or training, and perceived barriers were not independently associated with high acceptance.

The item-level findings revealed important gaps in public understanding of appropriate antibiotic use and AMR. Similar deficiencies in public knowledge have been reported in previous studies conducted in Saudi Arabia and South Korea [27,28]. In contrast, a study conducted in Jordan reported a higher level of knowledge regarding antibiotics and AMR [29]. Differences between studies may reflect variation in questionnaire content, scoring methods, participant characteristics, previous exposure to public-health campaigns, and sampling strategies. Because the present questionnaire did not formally measure validated Technology Acceptance Model or Unified Theory of Acceptance and Use of Technology constructs [30,31], the observed knowledge gaps should not be interpreted through TAM or UTAUT or assumed to increase the perceived usefulness of SHTs. Instead, the findings indicate a continuing need for accurate, accessible, and sustained public education regarding antibiotic indications, treatment completion, non-prescription use, sharing, and the mechanisms of antimicrobial resistance.

Although most respondents reported completing prescribed antibiotic courses, 37.8% reported lifetime non-prescription antibiotic use and 31.3% reported having shared antibiotics with family members or friends. These findings are broadly consistent with previous studies from Saudi Arabia, Kuwait, and Pakistan that documented self-medication, incomplete treatment courses, and sharing or reuse of antibiotics [32,33,34]. However, the present questionnaire asked whether participants had ever used antibiotics without a prescription and did not determine when the behavior occurred, how frequently it occurred, or how the antibiotics were obtained. Accordingly, the finding cannot be interpreted as direct evidence that current prescription-only dispensing regulations are ineffective. The reported behavior may have occurred before regulatory enforcement or may have involved leftover antibiotics, medication sharing, purchase outside Saudi Arabia, or other sources. The coexistence of reported treatment completion with other inappropriate practices suggests that antibiotic-use behavior may be influenced by multiple factors, including cultural norms, perceived disease severity, access to healthcare, medication availability, and previous personal experience [35,36,37,38]. These findings support multilayered stewardship strategies that combine sustained education with regulation, professional guidance, surveillance, and infection-prevention measures.

Awareness and previous use of the evaluated digital tools were limited, with 30.4% reporting awareness of antibiotic-related mobile health applications and 37.6% reporting previous use of a digital medication-reminder tool. In contrast, 61.8% stated that they would be willing to use an antibiotic-reminder application, and 87.3% preferred electronic prescriptions. This difference highlights the need to distinguish awareness, previous use, stated willingness, and preference because these measures do not represent the same stage of technology adoption. Preference for electronic prescriptions may also reflect familiarity with existing healthcare infrastructure rather than voluntary adoption of an antibiotic-management application.

The mean acceptance score was 4.39 ± 0.66 on a five-point scale, and 79.7% of respondents met the predefined threshold for high stated acceptance. These findings indicate favorable attitudes toward the selected digital functions assessed in the questionnaire, including medication reminders, digital adherence support, trust in mobile health applications, and integration into public-health campaigns. However, they do not demonstrate actual application downloads, sustained engagement, implementation readiness, improved adherence, reduced inappropriate antibiotic use, or effects on antimicrobial resistance. Public acceptance of digital health interventions may exceed actual adoption because uptake can be influenced by usability, digital literacy, privacy concerns, cost, access, trust, and integration with healthcare services [16,39,40]. Prospective implementation studies are therefore required to determine whether favorable survey responses translate into sustained and effective use.

Women had a slightly higher mean acceptance score than men, although the continuous-score comparison narrowly missed conventional statistical significance. In the binary analysis, women had higher crude and adjusted odds of high acceptance. This finding is broadly consistent with some studies reporting greater use of health information resources and favorable attitudes toward selected digital-health services among women [41,42,43,44]. Nevertheless, the result should be interpreted cautiously. The overall multivariable model was not statistically significant and demonstrated only modest discrimination. Furthermore, the study did not measure potential explanatory factors such as digital literacy, previous health-technology experience, caregiving responsibilities, health consciousness, or perceived usefulness. Therefore, female sex should not be interpreted as a definitive or causal predictor of technology adoption. Previous Saudi research has similarly highlighted persistent gaps in public knowledge and practices regarding antibiotic use and antimicrobial resistance [45]. Successful digital transformation also depends on addressing organizational, technological, and implementation-related challenges [46].

Perceived barriers were not associated with acceptance in either the bivariate or adjusted analyses. The barrier count showed essentially no correlation with the continuous acceptance score and was not independently associated with high acceptance. Therefore, the findings do not support the interpretation that a greater number of perceived barriers increases willingness to adopt SHTs.

The findings have potential implications for community antimicrobial stewardship and digital-health research in Saudi Arabia. Selected SHTs may complement conventional public education by providing individualized reminders, timely antibiotic-related information, adherence support, bidirectional communication, and integration with electronic prescribing or healthcare platforms. However, they should not be presented as replacements for existing national AMR measures. Their potential role should be considered alongside prescription regulation, antimicrobial-consumption and resistance surveillance, hospital antimicrobial-stewardship programmes, professional education, infection prevention and control, and sustained community education.

The study did not evaluate participants’ previous exposure to national AMR campaigns or existing educational interventions. Consequently, it cannot determine whether the observed knowledge gaps reflect insufficient campaign reach, ineffective content, regional inequalities, differences in previous exposure, or other factors. Future studies should directly assess awareness of national AMR initiatives and determine whether previous educational exposure is associated with knowledge, antibiotic-use practices, and attitudes toward digital interventions.

The favorable stated attitudes observed in this online sample may support further evaluation of selected digital interventions, but they do not justify immediate national implementation. Before wider adoption, digital tools should undergo stakeholder co-design, usability testing, privacy and cybersecurity assessment, accessibility and digital-equity evaluation, integration testing with existing healthcare systems, and prospective assessment of actual behavioral and clinical outcomes. Particular attention should be given to older adults, individuals with limited digital literacy, socioeconomically disadvantaged groups, and residents of underrepresented or geographically remote regions.

Several limitations should be considered when interpreting the findings. First, the study used an online convenience sample, and approximately three-quarters of participants resided in the central region. The sample also included a high proportion of Saudi nationals and university-educated respondents. These characteristics limit national representativeness and may have resulted in the underrepresentation of residents from other regions and individuals with limited internet access or digital competence. Second, the study relied on self-reported responses and was therefore subject to recall and social-desirability bias. Participants may have overreported desirable practices, such as treatment completion, or underreported inappropriate antibiotic use.

Third, the cross-sectional design prevents conclusions regarding temporality or causality. Associations between gender and high acceptance cannot establish that gender itself determines acceptance or actual adoption. Fourth, the exclusive use of an online questionnaire may have introduced digital-selection bias, which is particularly relevant because the main outcome concerned attitudes toward digital technologies. Respondents willing and able to complete an online survey may already be more comfortable with digital tools, potentially inflating favorable attitudes.

Fifth, the questionnaire did not undergo documented formal forward–backward translation, external content-validity assessment, construct-validity testing, or domain-specific reliability assessment. The questionnaire also did not formally measure TAM or UTAUT constructs. These measurement limitations should be considered when interpreting the composite acceptance score. Sixth, the study measured hypothetical willingness and stated acceptance rather than actual adoption, sustained use, adherence, changes in antibiotic consumption, clinical outcomes, or AMR outcomes. Finally, the questionnaire did not assess the timing or source of non-prescription antibiotic use, prior exposure to national AMR campaigns, income, chronic-disease status, or presence of children, and these potentially relevant factors could not be included in the analyses.

Future research should use probability-based or mixed-mode recruitment, including online, paper-based, telephone, and interviewer-assisted approaches, to improve geographic and digital inclusion. Longitudinal and prospective implementation studies are also needed to assess actual uptake, sustained engagement, usability, digital equity, behavioral effects, and the effectiveness of selected SHTs as complementary components of antimicrobial-stewardship programmes.

## 5. Conclusions

In the present study, we assessed public knowledge and practices related to antibiotic use and antimicrobial resistance, together with awareness, perceived barriers, and stated acceptance of selected smart health technologies among adults residing in Saudi Arabia. The findings showed that important knowledge gaps and inappropriate antibiotic-use practices persisted, while the surveyed online respondents expressed generally favorable stated attitudes toward selected digital tools.

Exploratory multivariable analyses did not provide sufficiently strong predictive evidence to draw firm conclusions regarding the demographic determinants of high stated acceptance. Perceived barriers were not associated with acceptance, and no significant associations were observed for age group, educational level, or healthcare-related education or training.

These findings should not be interpreted as evidence of actual technology adoption, national implementation readiness, or intervention effectiveness. Because the study used an online convenience sample that was geographically concentrated in the central region, the results should not be generalized to the Saudi population as a whole.

Selected smart health technologies may complement, rather than replace, existing antimicrobial-stewardship measures by supporting medication reminders, antibiotic-related education, adherence, and integration with electronic healthcare services. Future nationally representative and prospective implementation studies are needed to evaluate usability, accessibility, digital equity, sustained adoption, privacy and cybersecurity, and effects on antibiotic-use behavior and antimicrobial resistance.

## Figures and Tables

**Table 1:** Descriptive statistics of the verified composite measures (N = 434).

Measure	Number of Items	Minimum	Maximum	Mean	SD
Barrier count	5	0	5	1.04	1.06
Acceptance score	4	1.25	5.00	4.39	0.66

Note: The barrier count represented the number of selected barriers, with “nothing” coded as 0. The acceptance score was calculated as the mean of four items coded from 1 (“strongly disagree”) to 5 (“strongly agree”). Higher scores indicated greater stated acceptance.

**Table 2:** Sociodemographic characteristics of the study participants (N = 434).

Characteristic	Category	n	%
Age group	18–24 years	89	20.5
	25–35 years	57	13.1
	36–45 years	106	24.4
	46–55 years	90	20.7
	≥56 years	92	21.2
Gender	Male	281	64.7
	Female	153	35.3
Nationality	Saudi	421	97.0
	Non-Saudi	13	3.0
Region of residence	Central	321	74.0
	Western	54	12.4
	Eastern	42	9.7
	Northern	10	2.3
	Southern	7	1.6
Educational level	Bachelor’s degree	227	52.3
	High school graduate	72	16.6
	Diploma	66	15.2
	Postgraduate degree	54	12.4
	Less than high school	15	3.5
Employment status	Employed, non-health sector	187	43.1
	Unemployed	140	32.3
	Student	77	17.7
	Employed, health sector	30	6.9
Healthcare-related education or training	No	301	69.4
	Yes	133	30.6

Note: Percentages may not total 100% because of rounding.

**Table 3:** Knowledge of antibiotics and antimicrobial resistance (N = 434).

Item and Response	n	%
Definition of antibiotic resistance		
Antibiotics stop working effectively against the microorganism	161	37.1
The human body becomes resistant to antibiotics	136	31.3
Antibiotics become stronger and more effective over time	39	9.0
Not sure	98	22.6
Factors that may cause antibiotic resistance ^a^		
Overusing antibiotics	299	68.9
Not completing the prescribed antibiotic course	247	56.9
Using antibiotics for viral infections	146	33.6
Sharing leftover antibiotics	90	20.7
Not sure	45	10.4
Organisms against which antibiotics were considered effective ^a^		
Bacteria	295	68.0
Viruses	173	39.9
Fungi	108	24.9
Parasites	77	17.7
Not sure	58	13.4
Can unnecessary antibiotic use lead to microbial resistance?		
Yes	275	63.4
No	50	11.5
Not sure	109	25.1
Is it safe to stop taking antibiotics when symptoms improve?		
No	316	72.8
Yes	82	18.9
Not sure	36	8.3
Do you think antibiotic resistance is increasing?		
Yes	351	80.9
No	83	19.1
Antibiotic resistance occurs when:		
Microorganisms become resistant to antibiotics	198	45.6
The human body becomes resistant to antibiotics	167	38.5
Not sure	69	15.9
Factors that may contribute to antibiotic resistance ^a^		
Not completing the full course	325	74.9
Taking antibiotics without a prescription	272	62.7
Using leftover antibiotics	137	31.6
Poor infection control	74	17.1
Not sure	51	11.8

Note: ^a^ Participants could select more than one response; therefore, percentages may exceed 100%. Percentages were calculated using all 434 respondents as the denominator.

**Table 4:** Practices related to antibiotic use (N = 434).

Variable	Response	n	%
Ever taken antibiotics without a physician’s prescription	No	270	62.2
	Yes	164	37.8
Always complete the prescribed antibiotic course	No	90	20.7
	Yes	344	79.3
Ever shared antibiotics with family or friends	No	298	68.7
	Yes	136	31.3
Usual sources of antibiotic-related information ^a^	Physician	367	84.6
	Pharmacist	214	49.3
	Internet or social media	104	24
	Friends or relatives	66	15.2

Note: ^a^ Participants could select more than one source; therefore, percentages may exceed 100%. Percentages were calculated using all 434 respondents as the denominator.

**Table 5:** Awareness, previous use, and willingness to use selected smart health technologies (N = 434).

Variable	Response	n	%
Aware of mobile health applications that help track antibiotic use	No	302	69.6
	Yes	132	30.4
Previously used an application, SMS alert, or smart device as a medication reminder	No	271	62.4
	Yes	163	37.6
Willing to use an antibiotic-reminder application	No	53	12.2
	Yes	268	61.8
	Maybe	113	26.0
Prefer an electronic prescription over a paper prescription	No	55	12.7
	Yes	379	87.3

**Table 6:** Perceived barriers to using health technologies (N = 434).

Perceived Barrier ^a^	n	%
Lack of interest	121	27.9
Lack of awareness	111	25.6
Privacy or security concerns	91	21
Difficulty using technology	87	20
Cost	41	9.4
No listed barrier	165	38

Note: ^a^ Participants could select more than one response; therefore, percentages may exceed 100%. Percentages were calculated using all 434 respondents as the denominator. Participants selecting “nothing” received a barrier count of 0.

**Table 7:** Attitudes toward selected smart health technologies (N = 434).

Statement	Strongly Disagree, n (%)	Disagree, n (%)	Neutral, n (%)	Agree, n (%)	Strongly Agree, n (%)
The public should be educated about the correct use of antibiotics ^a^	3 (0.7)	1 (0.2)	15 (3.5)	50 (11.5)	365 (84.1)
Smart technologies could help reduce antibiotic misuse in the community	2 (0.5)	6 (1.4)	36 (8.3)	139 (32.0)	251 (57.8)
I support integrating smart health technologies into public-health campaigns	3 (0.7)	7 (1.6)	32 (7.4)	120 (27.6)	272 (62.7)
I would change how I use antibiotics if digital tools helped me track and improve adherence	6 (1.4)	10 (2.3)	70 (16.1)	131 (30.2)	217 (50.0)
I trust mobile health applications to provide reliable advice regarding antibiotic use	2 (0.5)	12 (2.8)	53 (12.2)	132 (30.4)	235 (54.1)

Note: ^a^ The public-education item was reported separately and was not included in the four-item acceptance score.

**Table 8:** Association between gender and stated acceptance of smart health technologies (N = 434).

Gender	n	Acceptance Score, Mean ± SD	Lower Acceptance, n (%)	High Acceptance, n (%)
Male	281	4.35 ± 0.68	67 (23.8)	214 (76.2)
Female	153	4.47 ± 0.60	21 (13.7)	132 (86.3)

Note: For the continuous acceptance score, Welch’s t = −1.96, df = 349.62, *p* = 0.051. For high acceptance, the crude odds ratio for women versus men was 1.97 (95% CI: 1.15–3.36; Fisher’s exact *p* = 0.013). High acceptance was defined as a mean score of ≥4.

**Table 9:** Association between age group and high acceptance of smart health technologies (N = 434).

Age Group	Lower Acceptance, n (%)	High Acceptance, n (%)	Total, n
18–24 years	24 (27.0)	65 (73.0)	89
25–35 years	11 (19.3)	46 (80.7)	57
36–45 years	17 (16.0)	89 (84.0)	106
46–55 years	16 (17.8)	74 (82.2)	90
≥56 years	20 (21.7)	72 (78.3)	92
Total	88 (20.3)	346 (79.7)	434

Note: χ^2^(4) = 4.15, *p* = 0.387. The comparison of continuous acceptance scores across age groups yielded Kruskal–Wallis H = 7.37, *p* = 0.117. High acceptance was defined as a mean acceptance score of ≥4.

**Table 10:** Association between educational level and high acceptance of smart health technologies (N = 434).

Educational Level	Lower Acceptance	High Acceptance	Total
	n (%)	n (%)	n
Less than high school	1 (6.7)	14 (93.3)	15
High school graduate	18 (25.0)	54 (75.0)	72
Diploma	10 (15.2)	56 (84.8)	66
Bachelor’s degree	46 (20.3)	181 (79.7)	227
Postgraduate degree	13 (24.1)	41 (75.9)	54
Total	88 (20.3)	346 (79.7)	434

Note: High acceptance was defined as a mean acceptance score of ≥4. Chi-square test: χ^2^(4) = 4.27, *p* = 0.371.

**Table 11:** Multivariable logistic regression of factors associated with high stated acceptance (N = 434).

Predictor	Adjusted OR	95% CI	*p*-Value
Age group			
18–24 years	Reference		
25–35 years	1.29	0.54–3.06	0.567
36–45 years	1.66	0.77–3.60	0.197
46–55 years	1.7	0.77–3.74	0.191
≥56 years	1.49	0.68–3.26	0.321
Gender			
Male	Reference		
Female	1.88	1.04–3.40	0.036
Educational level			
Bachelor’s degree	Reference		
High school graduate	0.9	0.46–1.79	0.773
Diploma	1.44	0.67–3.08	0.351
Postgraduate degree	0.89	0.42–1.85	0.751
Less than high school	3.44	0.43–27.47	0.243
Healthcare-related education or training			
No	Reference		
Yes	0.95	0.56–1.61	0.847
Barrier count, per additional barrier	1	0.80–1.25	0.977

Note: High acceptance was defined as a mean acceptance score of ≥4. AOR, adjusted odds ratio; CI, confidence interval. Hosmer–Lemeshow χ^2^ = 4.93, df = 8, *p* = 0.765; AUC = 0.631; likelihood-ratio *p* = 0.274; pseudo-R^2^ = 0.030.

## Data Availability

The de-identified data supporting the findings of this study and the corresponding analytic code are available from the corresponding author upon reasonable request, subject to applicable ethical and institutional requirements. The data are not publicly available because of privacy and ethical restrictions.

## Supplementary Materials

The following supporting information can be downloaded at: https://www.mdpi.com/article/10.3390/healthcare14162480/s1, File S1: Bilingual Questionnaire Used in the Study.
