# Supplementary material for: Public Knowledge, Practices, and Acceptance of Smart Health Technologies Addressing Antibiotic Use and Resistance in Saudi Arabia
Source: Healthcare (Basel). 2026 Aug 11;14(16):2480. doi: 10.3390/healthcare14162480 (PMC13512551; doi:10.3390/healthcare14162480)
Supplement: Supplementary file 1 [file healthcare-14-02480-s001.zip › healthcare-4456379-supplementary.pdf]

Supplementary Material S1

Bilingual Questionnaire Used in the Study

Public Knowledge, Practices, and Acceptance of Smart Health Technologies Addressing Antibiotic Use and Resistance in Saudi Arabia

This file reproduces the survey questions and response options without participant data. Item numbering has been standardized for clarity.

Participant Consent | موافقة المشارك

**C1. I am at least 18 years old, reside in Saudi Arabia, and voluntarily agree to participate.**	**C1. أبلغ من العمر 18 عامًا أو أكثر، وأقيم في المملكة العربية السعودية، وأوافق طوعًا على المشاركة.**
☐ I agree to participate<br>☐ I do not agree to participate	أوافق على المشاركة ☐<br>لا أوافق على المشاركة ☐

Section A. Sociodemographic Characteristics | القسم أ. الخصائص الديموغرافية

**1. Age**	**1. العمر**
☐ 18–24 years<br>☐ 25–35 years<br>☐ 36–45 years<br>☐ 46–55 years<br>☐ ≥56 years	18–24 سنة ☐<br>25–35 سنة ☐<br>36–45 سنة ☐<br>46–55 سنة ☐<br>56 سنة فأكثر ☐

**2. Gender**	**2. الجنس**
☐ Male<br>☐ Female	ذكر ☐<br>أنثى ☐

**3. Nationality**	**3. الجنسية**
☐ Saudi<br>☐ Non-Saudi	سعودي ☐<br>غير سعودي ☐

**4. Region of residence**	**4. منطقة الإقامة**
☐ Central<br>☐ Western<br>☐ Eastern<br>☐ Northern<br>☐ Southern	الوسطى ☐<br>الغربية ☐<br>الشرقية ☐<br>الشمالية ☐<br>الجنوبية ☐

**5. Educational level**	**5. المستوى التعليمي**
☐ Less than high school<br>☐ High school graduate<br>☐ Diploma<br>☐ Bachelor's degree<br>☐ Postgraduate degree	أقل من الثانوية ☐<br>خريج الثانوية ☐<br>دبلوم ☐<br>بكالوريوس ☐<br>دراسات عليا ☐

**6. Employment status**	**6. الحالة الوظيفية**

☐ Student<br>☐ Employed (health sector)<br>☐ Employed (non-health sector)<br>☐ Unemployed	طالب ☐<br>موظف في القطاع الصحي ☐<br>موظف في قطاع غير صحي ☐<br>غير موظف ☐

**7. Have you received formal education or training in a healthcare-related field?**	**7. هل تلقيت تعليمًا أو تدريبًا رسميًا في مجال متعلق بالرعاية الصحية؟**
☐ Yes<br>☐ No	نعم ☐<br>لا ☐

Section B. Knowledge of Antibiotics and Antimicrobial Resistance | القسم ب. المعرفة بالمضادات الحيوية ومقاومة مضادات الميكروبات

**8. Which statement best describes antibiotic resistance?**	**8. أي عبارة تصف مقاومة المضادات الحيوية بشكل أفضل؟**
☐ Antibiotics stop working effectively against the microorganism<br>☐ The human body becomes resistant to antibiotics<br>☐ Antibiotics become stronger and more effective over time<br>☐ Not sure	تتوقف المضادات الحيوية عن العمل بفعالية ضد الكائن الممرض ☐<br>يصبح جسم الإنسان مقاومًا للمضادات الحيوية ☐<br>تصبح المضادات الحيوية أقوى وأكثر فعالية بمرور الوقت ☐<br>لست متأكدًا ☐

**9. Which factors may cause antibiotic resistance?**<br>(Select all that apply)	**9. ما العوامل التي قد تسبب مقاومة المضادات الحيوية؟**<br>(اختر كل ما ينطبق)
☐ Overusing antibiotics<br>☐ Not completing the prescribed antibiotic course<br>☐ Using antibiotics for viral infections<br>☐ Sharing leftover antibiotics<br>☐ Not sure	الإفراط في استخدام المضادات الحيوية ☐<br>عدم إكمال دورة المضاد الحيوي الموصوفة ☐<br>استخدام المضادات الحيوية للعدوى الفيروسية ☐<br>مشاركة المضادات الحيوية المتبقية ☐<br>لست متأكدًا ☐

**10. Antibiotics are effective mostly against:**<br>(Select all that apply)	**10. المضادات الحيوية فعالة غالبًا ضد:**<br>(اختر كل ما ينطبق)
☐ Bacteria<br>☐ Viruses<br>☐ Fungi<br>☐ Parasites<br>☐ Not sure	البكتيريا ☐<br>الفيروسات ☐<br>الفطريات ☐<br>الطفيليات ☐<br>لست متأكدًا ☐

**11. Can unnecessary antibiotic use lead to microbial resistance?**	**11. هل يمكن أن يؤدي الاستخدام غير الضروري للمضادات الحيوية إلى المقاومة الميكروبية؟**
☐ Yes<br>☐ No<br>☐ Not sure	نعم ☐<br>لا ☐<br>لست متأكدًا ☐

**12. Is it safe to stop taking antibiotics when symptoms improve?**	**12. هل من الآمن التوقف عن تناول المضادات الحيوية عند تحسن الأعراض؟**
☐ Yes<br>☐ No<br>☐ Not sure	نعم ☐<br>لا ☐<br>لست متأكدًا ☐

13. Do you think antibiotic resistance is increasing?	13. هل تعتقد أن مقاومة المضادات الحيوية تتزايد؟
☐ Yes<br>☐ No	نعم ☐<br>لا ☐

14. Antibiotic resistance occurs when:	14. تحدث مقاومة المضادات الحيوية عندما:
☐ Microorganisms become resistant to antibiotics<br>☐ The human body becomes resistant to antibiotics<br>☐ Not sure	تصبح الكائنات الدقيقة مقاومة للمضادات الحيوية ☐<br>يصبح جسم الإنسان مقاومًا للمضادات الحيوية ☐<br>لست متأكدًا ☐

15. Which factors may contribute to antibiotic resistance?<br>(Select all that apply)	15. أي العوامل التالية قد تسهم في مقاومة المضادات الحيوية؟<br>(اختر كل ما ينطبق)
☐ Not completing the full course<br>☐ Taking antibiotics without a prescription<br>☐ Using leftover antibiotics<br>☐ Poor infection control<br>☐ Not sure	عدم إكمال دورة العلاج كاملة ☐<br>تناول المضادات الحيوية دون وصفة طبية ☐<br>استخدام المضادات الحيوية المتبقية ☐<br>ضعف مكافحة العدوى ☐<br>لست متأكدًا ☐

Section C. Antibiotic-Use Practices | القسم ج. ممارسات استخدام المضادات الحيوية

16. Have you ever taken antibiotics without a physician's prescription?	16. هل سبق لك تناول مضاد حيوي دون وصفة من الطبيب؟
☐ Yes<br>☐ No	نعم ☐<br>لا ☐

17. Do you always complete the prescribed antibiotic course?	17. هل تكمل دائمًا دورة المضاد الحيوي الموصوفة؟
☐ Yes<br>☐ No	نعم ☐<br>لا ☐

18. Have you ever shared antibiotics with family members or friends?	18. هل سبق لك مشاركة المضادات الحيوية مع أفراد الأسرة أو الأصدقاء؟
☐ Yes<br>☐ No	نعم ☐<br>لا ☐

19. What are your usual sources of antibiotic-related information?<br>(Select all that apply)	19. ما مصادر معلوماتك المعتادة حول المضادات الحيوية؟<br>(اختر كل ما ينطبق)
☐ Physician<br>☐ Pharmacist<br>☐ Internet or social media<br>☐ Friends or relatives	الطبيب ☐<br>الصيدلي ☐<br>الإنترنت أو وسائل التواصل الاجتماعي ☐<br>الأصدقاء أو الأقارب ☐

Section D. Awareness and Use of Smart Health Technologies | القسم د. الوعي واستخدام تقنيات الصحة الذكية

20. Are you aware of mobile health applications that help track antibiotic use?	20. هل تعرف تطبيقات صحية على الهاتف تساعد في متابعة استخدام المضادات الحيوية؟
☐ Yes<br>☐ No	نعم ☐<br>لا ☐

21. Have you previously used an application, SMS alert, or smart device as a medication reminder?	هل سبق لك استخدام تطبيق أو رسالة نصية أو جهاز ذكي كتذكير بالدواء؟ .21
☐ Yes<br>☐ No	نعم ☐<br>لا ☐

22. Would you be willing to use an application that reminds you to take antibiotics on schedule?	هل ترغب في استخدام تطبيق يذكّرك بتناول المضاد الحيوي في موعده؟ .22
☐ Yes<br>☐ No<br>☐ Maybe	نعم ☐<br>لا ☐<br>ربما ☐

23. Would you prefer an electronic prescription over a paper prescription if both were available?	هل تفضل الوصفة الإلكترونية على الوصفة الورقية إذا كان الخياران متاحين؟ .23
☐ Yes<br>☐ No	نعم ☐<br>لا ☐

Section E. Perceived Barriers | القسم هـ. العوائق المتصورة

24. What may prevent you from using health technologies? (Select all that apply)	ما الذي قد يمنعك من استخدام تقنيات الصحة؟ .24<br>(اختر كل ما ينطبق)
☐ Lack of interest<br>☐ Lack of awareness<br>☐ Privacy or security concerns<br>☐ Difficulty using technology<br>☐ Cost<br>☐ Nothing	عدم الاهتمام☐<br>نقص الوعي ☐<br>مخاوف الخصوصية أو الأمان ☐<br>صعوبة استخدام التقنية ☐<br>التكلفة ☐<br>لا يوجد عائق ☐

Section F. Attitudes toward Smart Health Technologies | القسم و. الاتجاهات نحو تقنيات الصحة الذكية

25. The public should be educated about the correct use of antibiotics.	يجب تثقيف الجمهور حول الاستخدام الصحيح للمضادات الحيوية. .25
☐ Strongly disagree<br>☐ Disagree<br>☐ Neutral<br>☐ Agree<br>☐ Strongly agree	لا أوافق بشدة ☐<br>لا أوافق ☐<br>محايد ☐<br>أوافق ☐<br>أوافق بشدة ☐

26. Smart technologies could help reduce antibiotic misuse in the community.	يمكن للتقنيات الذكية أن تساعد في تقليل سوء استخدام المضادات الحيوية في المجتمع. .26
☐ Strongly disagree<br>☐ Disagree<br>☐ Neutral<br>☐ Agree<br>☐ Strongly agree	لا أوافق بشدة ☐<br>لا أوافق ☐<br>محايد ☐<br>أوافق ☐<br>أوافق بشدة ☐

27. I support integrating smart health technologies into public-health campaigns.	أؤيد دمج تقنيات الصحة الذكية في حملات الصحة العامة. .27

☐ Strongly disagree<br>☐ Disagree<br>☐ Neutral<br>☐ Agree<br>☐ Strongly agree	☐ لا أوافق بشدة<br>☐ لا أوافق<br>☐ محايد<br>☐ أوافق<br>☐ أوافق بشدة

**28. I would change how I use antibiotics if digital tools helped me track and improve adherence.**	**28. سأغيّر طريقة استخدامي للمضادات الحيوية إذا ساعدتني الأدوات الرقمية في المتابعة وتحسين الالتزام.**
☐ Strongly disagree<br>☐ Disagree<br>☐ Neutral<br>☐ Agree<br>☐ Strongly agree	☐ لا أوافق بشدة<br>☐ لا أوافق<br>☐ محايد<br>☐ أوافق<br>☐ أوافق بشدة

**29. I trust mobile health applications to provide reliable advice regarding antibiotic use.**	**29. أثق في تطبيقات الصحة المتنقلة لتقديم نصائح موثوقة بشأن استخدام المضادات الحيوية.**
☐ Strongly disagree<br>☐ Disagree<br>☐ Neutral<br>☐ Agree<br>☐ Strongly agree	☐ لا أوافق بشدة<br>☐ لا أوافق<br>☐ محايد<br>☐ أوافق<br>☐ أوافق بشدة
